# The role of iron in *Mycobacterium smegmatis* biofilm formation: the exochelin siderophore is essential in limiting iron conditions for biofilm formation but not for planktonic growth

**DOI:** 10.1111/j.1365-2958.2007.05935.x

**Published:** 2007-10

**Authors:** Anil Ojha, Graham F Hatfull

**Affiliations:** Department of Biological Sciences, University of Pittsburgh Pittsburgh, PA 15260, USA

## Abstract

Many species of mycobacteria form structured biofilm communities at liquid–air interfaces and on solid surfaces. Full development of *Mycobacterium smegmatis* biofilms requires addition of supplemental iron above 1 μM ferrous sulphate, although addition of iron is not needed for planktonic growth. Microarray analysis of the *M. smegmatis* transcriptome shows that iron-responsive genes – especially those involved in siderophore synthesis and iron uptake – are strongly induced during biofilm formation reflecting a response to iron deprivation, even when 2 μM iron is present. The acquisition of iron under these conditions is specifically dependent on the exochelin synthesis and uptake pathways, and the strong defect of an iron–exochelin uptake mutant suggests a regulatory role of iron in the transition to biofilm growth. In contrast, although the expression of mycobactin and iron ABC transport operons is highly upregulated during biofilm formation, mutants in these systems form normal biofilms in low-iron (2 μM) conditions. A close correlation between iron availability and matrix-associated fatty acids implies a possible metabolic role in the late stages of biofilm maturation, in addition to the early regulatory role. *M. smegmatis* surface motility is similarly dependent on iron availability, requiring both supplemental iron and the exochelin pathway to acquire it.

## Introduction

Most bacteria are found in the environment not as isolated cells growing planktonically, but as biofilms – organized sessile communities of cells surrounded by an extracellular matrix that are attached either to surfaces or at liquid–air interfaces (Costerton *et al*., 1999; Davey and O'Toole, 2000; O'Toole *et al*., 2000; Hall-Stoodley *et al*., 2004). While the ability of *Mycobacterium tuberculosis*– one of human's greatest microbial enemies (Frieden and Driver, 2003) – to form biofilms remains unclear, many other species of mycobacteria, including *M. avium* (Carter *et al*., 2003), *M. fortuitum* (Hall-Stoodley and Lappin-Scott, 1998), *M. marinum* (Hall-Stoodley *et al*., 2006) and *M. smegmatis* (Recht *et al*., 2000), do so readily. Because mycobacterial infections are notoriously troublesome to treat effectively with antibiotics, a possible involvement of drug tolerance arising from mycobacterial biofilm formation cannot be eliminated (Teng and Dick, 2003). However, little is known about the molecular events involved in mycobacterial biofilm formation, the nature of the matrix, or the complexity of the biofilm structure.

The formation of bacterial biofilms involves a developmental process that begins with surface attachment, followed by spreading, maturation and matrix synthesis (O'Toole *et al*., 2000). This process is accompanied by changes in gene expression profiles, and these have been described for several prokaryotes, including *Escherichia coli* (Schembri *et al*., 2003; Beloin *et al*., 2004; Ren *et al*., 2004a; Pruss *et al*., 2006), *Pseudomonas aeruginosa* (Whiteley *et al*., 2001; Waite *et al*., 2005; 2006), *Bacillus subtilis* (Stanley *et al*., 2003; Ren *et al*., 2004b), *Vibrio cholerae* (Moorthy and Watnick, 2005), *Xylella fastidiosa* (de Souza *et al*., 2004; 2005), *Thermatoga maritima* (Pysz *et al*., 2004), *Staphylococcus aureus* (Beenken *et al*., 2004), *Campylobacter jejuni* (Sampathkumar *et al*., 2006) and *Streptococcus pyogenes* (Cho and Caparon, 2005). These studies revealed several key findings. First, bacterial biofilms are likely composed of heterogeneous populations of cells experiencing different microenvironments and possibly expressing different subsets of genes, and there are large variations in planktonic cells' growth conditions (Lazazzera, 2005). Second, there is no single core biofilm regulon present in these bacteria, although induction of stress responses is common (Beloin and Ghigo, 2005). Third, a substantial portion of genes differentially expressed in biofilms are also expressed in stationary-phase cells (Beloin and Ghigo, 2005). Fourth, genes of unknown function constitute a high proportion of genes differentially expressed in biofilms (Beloin and Ghigo, 2005). Lastly, different sets of genes are expressed at different stages throughout the course of biofilm development (Waite *et al*., 2005; Domka *et al*., 2007).

All mycobacterial species, including *Mycobacterium tuberculosis*, the causative agent of human tuberculosis, exhibit extraordinary survival ability in stressful conditions (McCune *et al*., 1966; Stewart *et al*., 2003; Gomez and McKinney, 2004), and while many species are known to form biofilms, little is known about either the genetic requirements, patterns of gene expression, or the nature of the extracellular matrix. In *M. ulcerans*, biofilm formation is implicated during the initial stages of infection in its insect host *Naucoris cimicoids* (Marsollier *et al*., 2005), and in *M. marinum*, biofilm formation correlates with a cording morphology (Hall-Stoodley *et al*., 2006). *M. smegmatis* forms pellicle-like biofilms at air–liquid interfaces that involve sliding motility (Recht *et al*., 2000; Recht and Kolter, 2001), and undecaprenyl phosphokinase is required for biofilm and smegma formation (Rose *et al*., 2004). *M. smegmatis* biofilm formation appears to be separable into early events that involve attachment and spreading, followed by the late processes of maturation and matrix formation, and a mutant defective in GroEL1 is specifically deficient in the late stages (Ojha *et al*., 2005). Furthermore, GroEL1 is required for a change in fatty acid synthesis during biofilm formation that results in the increased synthesis of C_56_–C_68_ fatty acids (Ojha *et al*., 2005), and because mycobacterial genomes do not contain the genetic apparatus for exopolysaccharide biosynthesis (Zambrano and Kolter, 2005), the matrix is probably primarily composed of fatty acids. *M. smegmatis* biofilm maturation also requires the presence of a 2 μM iron supplement, and the removal of this supplement – while having no effect on the planktonic growth – impairs the biofilm development (Ojha *et al*., 2005) – suggesting that iron uptake systems may play important roles in biofilm development.

Iron is an essential cofactor for most bacteria, although the acquisition of iron – especially by bacterial pathogens – poses a potential problem (De Voss *et al*., 1999; Rodriguez and Smith, 2003). The mycobacteria have solved this by synthesizing and secreting siderophores – high-affinity iron chelators – which are then taken up as siderophore–iron complexes (Yu *et al*., 1998; Rodriguez and Smith, 2003). Some mycobacteria, such as *M. smegmatis*, synthesize two different siderophores, a pentapeptide derivative called exochelin, and two forms of the salicylate-derivative mycobactin, an acylated cell-associated form and a more polar carboxymycobactin that is extracellular (De Voss *et al*., 1999). Other mycobacteria, such as *M. tuberculosis*, synthesize only the mycobactins, and these are critical for growth in iron-limiting conditions in macrophages and in mice (De Voss *et al*., 2000; Timm *et al*., 2003). Expression of the acquisition genes is typically tightly regulated and is induced under iron-limiting conditions. In *M. tuberculosis*, expression of over 150 open reading frames (ORFs) are responsive to iron depletion, about one-third of which – including the Rv2377c–Rv2386c mycobactin biosynthetic operon – are under control of the IdeR regulator (Rodriguez *et al*., 2002; Wisedchaisri *et al*., 2007); *M. smegmatis* siderophore biosynthesis – including exochelin – is also under IdeR control (Dussurget *et al*., 1996). The mycobacteria contain one or more FurA-family genes that are involved in metal uptake and have been shown to respond to oxidative stress (Milano *et al*., 2001; Zahrt *et al*., 2001).

In this paper, we show that iron plays an essential and specific role in the formation of *M. smegmatis* biofilms. Addition of supplemental iron (2 μM) is required for biofilm formation, although when *M. smegmatis* biofilms develop under these conditions, there is substantial upregulation of the iron acquisition genes, including those involved in biosynthesis and uptake of siderophores. Mutational analysis shows that, while the exochelin biosynthesis and uptake systems are not required for planktonic growth, they are essential for biofilm formation under iron-limiting conditions, whereas the iron ABC transporters and mycobactin synthesis genes – although highly upregulated – are not. While the specific metabolic requirement for iron remains unclear, the availability of iron correlates closely with the synthesis of C_56_–C_68_ fatty acids that are key components of the *M. smegmatis* biofilm extracellular matrix.

## Results

### Regulation of iron responsive genes by transcriptome analysis of biofilms

**Fig. 1:**
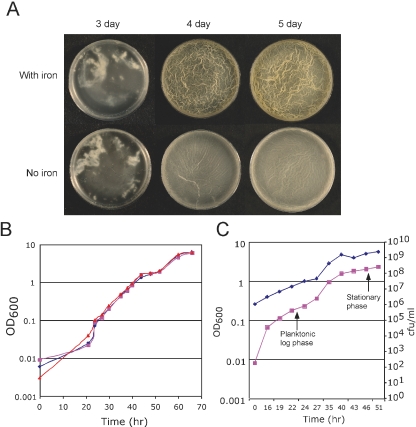
Biofilm and planktonically grown cells used for microarray analyses. A. Biofilm cultures of wild-type strain of *M. smegmatis* mc^2^155 were grown in a modified M63 medium, either with or without supplemental iron (2 μM FeSO_4_) as shown, for 3, 4 or 5 days. RNA samples for microarray analysis were harvested from the 3 and 4 day plates grown in iron-supplemented medium. B. Planktonic growth of wild-type *M. smegmatis* mc^2^155 in no iron (blue), 2 μM iron (purple) or 50 μM iron (red). C. Growth curves of *M. smegmatis* mc^2^155 in liquid medium showing the increase in cell density (OD_600_; purple) and viable colony counts (cfu ml^−1^; blue). Arrows indicate the points at which cells were harvested for the exponentially growing planktonic and stationary phase to prepare samples for microarray analysis.

The modified M63 medium that supports robust growth of pellicle-like *M. smegmatis* biofilms contains a relatively low supplemental iron concentration of 2 μM ferrous sulphate (Ojha *et al*., 2005). Inclusion of iron at this concentration is important for normal biofilm maturation, and omission leads to the inability to form mature biofilms, even though planktonic growth is unaffected (Ojha *et al*., 2005). In the absence of the iron supplement, surface growth is observed but without the reticulated appearance associated with formation of the extracellular matrix (Fig. 1A). Planktonic growth, however, is normal over a wide range of iron concentrations (0–50 μM), and because no specific addition of iron is required for planktonic growth, we assume that residual iron contaminants in other media components can satisfy any iron demands (Fig. 1B). Because these observations show that there is apparently a specific requirement for iron to support biofilm formation, we performed microarray analysis to determine how the iron regulons respond during biofilm development.

Dual-colour microarray experiments were performed to compare expression patterns at two different stages of biofilm growth and in early stationary phase, using a reference sample of exponentially growing planktonic cells (see Fig. 1C). The conditions used for growth of *M. smegmatis* biofilms were as described previously (Ojha *et al*., 2005) in which biofilms develop through distinct stages of visual appearance beginning at 3 days after inoculation with the formation of a thin film at the liquid–air interface, appearance of mature biofilm structures starting at 4 days, and complete maturation by 7 days. We chose to analyse expression after 3 and 4 days of biofilm growth on the assumption that these would represent prematuration (3 days) and maturation (4 days) phases, and would avoid additional regulatory effects that might occur once cells stop growing (beyond 7 days). For each of the three experimental comparisons (3 day biofilm, 4 day biofilm, stationary phase), RNA was isolated from three different cultures and used in separate hybridizations, one of which used the cy3/cy5 dyes in a flipped configuration. Whole-genome microarray slides [kindly provided by the Pathogen Functional Genomic Resource Center at the Institute for Genomic Research (TIGR); http://pfgrc.tigr.org/slide_html/array_descriptions/M_smegmatis_2.shtml] containing four duplicate spots of each of 6530 ORFs were used, and the fluorescence values were normalized according to the global intensity both within and across all slides as described previously (Yang *et al*., 2002). A complete dataset for the microarrays is deposited at Gene Expression Omnibus of National Center for Biotechnology Information (http://www.ncbi.nlm.nih.gov/geo) and can be accessed by accession number GPL4686; a summary of the data is included in Table S1. (It should be noted that the *M. smegmatis* genome annotation and corresponding microarray tables have been changed since this work was performed. The gene numbering used here follows the old annotation, MsmegXXXX, whereas the names in the newer annotation are represented as Msmeg_XXXX. The correspondence between the older and newer numbering systems is noted in the gene tables at http://www.tigr.org. One notable general finding was that under all of the conditions tested, only approximately one-half of all the *M. smegmatis* genes are expressed above background levels; not surprisingly, in planktonic growth the most highly expressed genes are primarily those involved in protein synthesis (Table S2). Additional validationof the microarray data was determined by performing real-time reverse transcription polymerase chain reaction (RT-PCR) evaluations on selected putative regulated genes (Fig. S1).

**Fig. 2:**
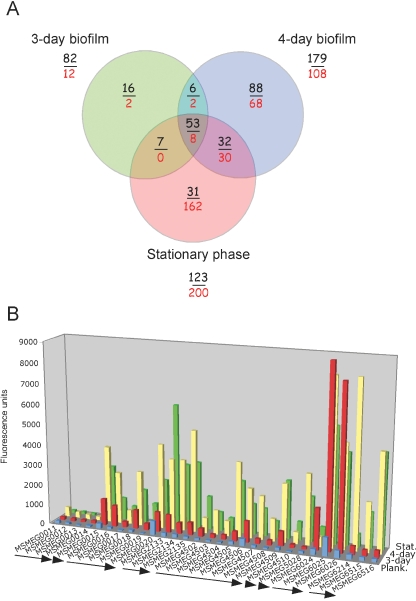
Schematic representations of transcriptome responses in 3 day biofilm, 4 day biofilm and stationary-phase conditions. A. Conditions corresponding to the 3 day biofilm (green circle), 4 day biofilm (blue circle) and stationary phase (red circle) are shown, with the numbers of induced genes (> 4-fold) shown in black and the number of repressed genes (> 4-fold) shown in red. The numbers of induced and regulated genes common to two or more conditions are included at the intersections of the three circles. A complete list of microarray data for genes in each category is included in Table S2. B. Induction of genes involved in iron acquisition during biofilm and stationary-phase growth. Twenty-nine genes arranged in nine operons (Msmeg0011–0014, Msmeg0015, Msmeg0016–0018, Msmeg2133–2135, Msmeg4502–4509, Msmeg4510, Msmeg5028, Msmeg6024–6026 and Msmeg6214–6216) putatively involved in iron acquisition show substantial levels of induction after 4 days of biofilm development. Arrows indicate the operon arrangements. Average fluorescence units are shown for the exponential planktonic (blue bars), 3 day biofilm (red bars), 4 day biofilm (yellow bars) and stationary phase (green bars). Fluorescence values for the exponential planktonic sample are the average of 12 normalized slides, and for the other three experimental conditions, are the average from three independent experiments.

The first question we wished to address was whether there were global changes in gene expression during *M. smegmatis* biofilm formation. We observe that there are relatively modest changes in the 3 day biofilm sample, with only ∼1.5% genes changing expression by four-fold or more (see Fig. S2). This pattern changes substantially by day 4 of biofilm growth, where ∼4.5% of the genome changes by fourfold for more (Fig. S2); a similar proportion of genes (approximately 4.9% of the genome) change expression in stationary phase (Fig. S2). In general, the majority of genes in the stationary-phase sample are repressed, while those in the biofilm samples are induced; many genes respond to two or more of the conditions tested. An illustration of these patterns is shown in Fig. 2A, and a list of genes is provided in Table S3.

While the complete set of iron-responsive genes in *M. smegmatis* has not been determined, those involved in siderophore biosynthesis and uptake have been described (Fiss *et al,* 1994), and other iron-responsive genes have been identified by microarray analysis of *M. tuberculosis* (Rodriguez *et al*., 2002). Because each of our samples was grown in the presence of 2 μM iron – which is considered to be a low-iron condition for *M. tuberculosis* (Rodriguez *et al*., 2002) – we anticipated that all or many of the iron-responsive genes would be expressed at moderate or relatively high levels. However, in the planktonic exponentially growing sample, while some expression of the siderophore biosynthetic genes such as Msmeg0015–0019 (exochelins) and Msmeg4502–4510 (mycobactins) is observed, it is at a low level, suggesting that under these conditions *M. smegmatis* is not responding to any substantial iron deficiency (Fig. 2B). In the 3 day biofilm sample, there is only modest change in expression (3- to 4-fold induction) of the mycobactin biosynthetic genes (Msmeg4502–4510), and a somewhat larger (5- to 10-fold) induction of the exochelin biosynthetic genes (Msmeg0015–0019). However, there is also substantial induction (> 10-fold) of a putative iron ABC transport system (Msmeg6024–6026), and the absolute level of expression in the 3 day biofilm sample is higher than any of the other putative iron-responsive operons (Fig. 2B). Interestingly these genes are located immediately downstream of a four-gene operon (Msmeg6027–6030) encoding ribosomal protein subunits (S18, S14, L33 and L28 respectively) that are also highly induced (> 100-fold) at this stage (Table S1).

In the 4 day biofilm sample, the induction of putative iron-regulated genes is much more pronounced (Fig. 2B), including both the exochelin and mycobactin biosynthetic operons. There is also modest induction (3- to 4-fold) of the exochelin uptake genes (Msmeg0011–0014), although the expression level is lower than the biosynthetic genes. We also observe substantial induction of the Msmeg2133–2135 operon that encodes homologues of the *M. tuberculosis* functions proposed to acylate mycobactins (LaMarca *et al*., 2004; Krithika *et al*., 2006), Msmeg6214 that encodes a FurA-family member, and the Msmeg6515–6516 operon encoding a putative metal ABC transporter (Fig. 2B). The induction of the FurA-like protein (Msmeg6214) is somewhat surprising because the well-characterized FurA proteins (including the one encoded by Msmeg3466) typically act as transcriptional repressors (Zahrt *et al*., 2001). While Msmeg6214 could be downregulating genes in the 4 day biofilm sample, it is not induced in the stationary-phase sample, and we cannot exclude the possibility that it acts as a biofilm-specific activator. We note that none of the other three putative FurA-family regulators in *M. smegmatis* (Msmeg3466, Msmeg4482 and Msmeg6345) are substantially induced in any of the growth conditions tested here. Finally, we note that many of the iron-responsive genes are induced in the stationary-phase sample, and we conclude that under the growth conditions used, both the stationary-phase and biofilm samples are responding to substantial iron deprivation.

### The exochelin uptake system is required for biofilm formation in iron-limiting conditions

**Fig. 3:**
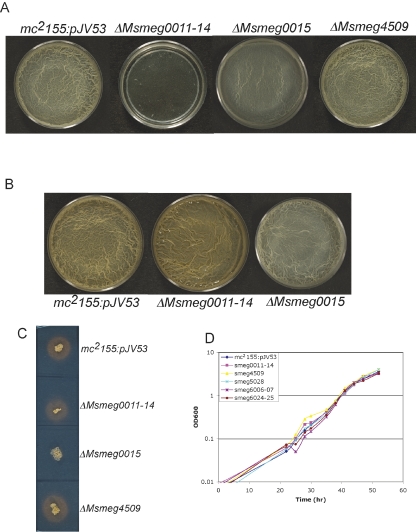
Ferric exochelin biosynthesis (Msmeg0015) and uptake genes (Msmeg0011–0014) are required for biofilm formation but not for planktonic growth. A. Mutants defective in exochelin biosynthesis (ΔMsmeg0015, *fxbA*), exochelin uptake (ΔMsmeg0011–0014, *fxuABC*) and mycobactin biosynthesis (ΔMsmeg4509, *mbtB*) were generated by recombineering (van Kessel and Hatfull, 2007) and tested for biofilm growth. Growth after 4 days of biofilm development is shown using standard media containing a 2 μM iron supplement. All strains, including the wild-type control (mc^2^155), carry the pJV53 recombineering plasmid. B. Suppression of the biofilm defect of the mutants ΔMsmeg0011–0014 and ΔMsmeg0015 by addition of 50 μM iron to the growth medium. C. CAS-agar assay to test the synthesis and secretion of siderophore by ΔMsmeg0011–0014, ΔMsmeg0015, ΔMsmeg4509 and *mc*^*2*^*155: pJV53* was used as a control. The orange halo around the colony is indicative of siderophore-mediated chelation of iron from CAS–iron complexes. D. Planktonic growth of the mutants described in A, compared with the parental *M. smegmatis* mc^2^155 strain. Cultures were grown in biofilm medium containing a 2 μM iron supplement.

The requirement for the addition of 2 μM iron along with the induction of iron-acquisition genes suggests that iron plays a role in biofilm formation that is distinct from that in planktonic growth. It thus seems likely that one or more of the iron-acquisition systems play a critical role in biofilm formation. To test this, we constructed a series of *M. smegmatis* mutants in which we deleted those genes involved in siderophore synthesis or other putative iron uptake systems, and examined their ability to form mature biofilms. Interestingly, mutants defective in mycobactin biosynthesis (ΔMsmeg4509; *mbtB*), iron ABC transporters (ΔMsmeg6006–6007; ΔMsmeg6024–6025) or the iron-utilization gene ΔMsmeg5028 have similar biofilm growth as the parent *M. smegmatis* strain (Table 1; Fig. 3). Thus, even though these genes are all strongly upregulated in the 4 day biofilm, none are required for biofilm maturation under these conditions. In contrast, mutants defective in either ferric exochelin biosynthesis, Δ*fxbA* (ΔMsmeg0015), or uptake Δ*fxuABC* (ΔMsmeg0011–0014) are strongly defective in biofilm formation; loss of the exochelin uptake system has the strongest biofilm defect among all the mutants we have analysed (Table 1) – including the previously described Δ*groEL1* mutant (Ojha *et al*., 2005) – whereas the mutant defective in exochelin biosynthesis has a relatively milder defect (and behaves similarly to the Δ*groEL1* mutant) (Table 1; Fig. 3A). However, both the exochelin synthesis and uptake mutants grow normally in planktonic growth in the presence of 2 μM iron (see Fig. 3D), as well as without addition of any supplemental iron (data not shown). We have constructed and tested seven additional mutants (ΔMsmeg0396, ΔMsmeg0923, ΔMsmeg1739, ΔMsmeg3411, ΔMsmeg5088, ΔMsmeg6030 and ΔMsmeg6214) that are defective in biofilm-regulated genes, most of which form normal biofilms; only the ΔMsmeg6030 mutant is mildly defective (Table 1). These observations are consistent with the interpretation that much of the global change in gene expression patterns is in response to the changing environment cells experience within a biofilm, and that few of these are involved in the biofilm-development pathway directly. The exochelin biosynthesis and uptake genes are clearly thus distinct in being not only induced but also required for biofilm growth.

**Table 1:** Properties of mutants defective in biofilm-regulated genes.

Strain	Putative functional role	4 day biofilm biomass (g per 10 ml)	Reduction (% wt)	Reference
mc^2^155:pJV53	Control	0.84 (±0.014)	100	This study
ΔMsmeg0011–0014	Exochelin uptake (*fxuABC*)	0.08 (±0.005)	9.5	This study
ΔMsmeg0015	Exochelin biosynthesis (f*xbA*)	0.18 (±0.018)	21.4	This study
ΔMsmeg0396	Fatty acid degradation (*fadE5*)	0.84 (±0.004)	100	This study
ΔMsmeg0923	Putative repressor	0.77 (±0.043)	91.7	This study
ΔMsmeg 1739	Oxidoreductase	0.85 (±0.007)	101	This study
ΔMsmeg3411	Putative HNH endonuclease	0.79 (±0.019)	94	This study
ΔMsmeg4509	Mycobactin biosynthesis (*MtbB*)	0.80 (±0.069)	95.2	This study
ΔMsmeg5028	Iron-utilization protein	0.84 (±0.008)	100	This study
ΔMsmeg5088	ABC transporter	0.77 (±0.020)	91.7	This study
ΔMsmeg6006–6007	Iron ABC transporter	0.88 (±0.022)	105	This study
ΔMsmeg6024–6025	Haem ABC transporter	0.88 (±0.041)	105	This study
ΔMsmeg6030	Ribosomal protein L28	0.40 (±0.040)	47.6	This study
ΔMsmeg6214	FurA-family protein	0.72 (±0.034)	85.7	This study
ΔgroEL1	Hsp60 chaperonin	0.23 (±0.006)	27.4	Ojha *et al*. (2005)

The strong and specific defects observed with the ΔMsmeg0011–0014 and ΔMsmeg0015 mutants suggest that the exochelin pathway is the dominant mechanism for acquiring the iron needed for biofilm development. To confirm that the defects observed are specifically related to iron acquisition, we tested whether addition of iron at a high concentration (50 μM) would suppress the mutant defects. As shown in Fig. 3B, addition of 50 μM iron substantially suppresses the mutant phenotype, and the biomass accumulated after 4 days of biofilm growth is similar to that of the parent strain (data not shown); presumably either the mycobactin- or other iron-acquisition systems are involved in iron uptake at these higher concentrations. We also tested the mutants defective in genes required for siderophore synthesis (ΔMsmeg0015, Δ*fxbA*; ΔMsmeg4509, Δ*mbtB*) and uptake (ΔMsmeg0011–0014, Δ*fxuABC*) in a CAS-agar assay (Fiss *et al*., 1994), and found that only the ΔMsmeg0015 (Δ*fxbA*) exochelin biosynthesis mutant has a clear defect in siderophore production (Fig. 3C).

The microarray data show that the *fxuABC* operon (Msmeg0011–0014) is expressed at a low level in the planktonic reference sample and is upregulated during biofilm development, although the levels of expression are substantially less than in the exochelin (*fxb*) and mycobactin (*mbt*) biosynthetic operons (Fig. 2B). To verify these responses and to examine the regulation of these operons under different iron conditions, we compared the expression patterns of three representative genes involved in exochelin uptake, *fxuC* (Msmeg0012), exochelin synthesis, *fxbA* (Msmeg0015) and mycobactin synthesis, *mbtB* (Msmeg4509) using real-time RT-PCR.

**Fig. 4:**
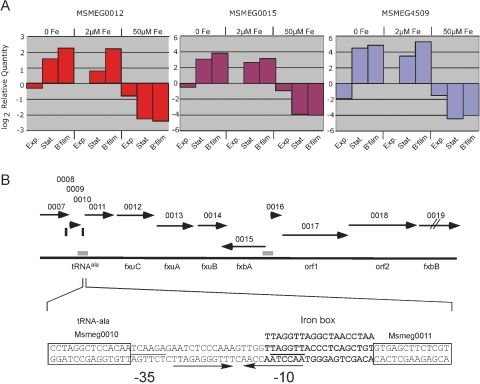
Regulation of genes involved in siderophore synthesis and uptake. A. Analysis of expression of exochelin biosynthesis, *fxbA* (Msmeg0015), exochelin uptake, *fxuC* (Msmeg0012) and mycobactin synthesis, *mbtB* (Msmeg4509) genes by real-time RT-PCR. Expression levels were measured in samples grown for 3 or 4 days under biofilm conditions, and in early stationary phase, and represented as the relative log_2_ change in expression level compared with early planktonic growth. Similar samples were tested under the same growth conditions but in the presence of either 0, 2 or 50 μM iron as shown. B. Organization of the exochelin synthesis and uptake genes. The positions of gene between Msmeg0007 and Msmeg0019 are shown, along with their gene designations; Msmeg0008 and Msmeg0010 encode tRNA^ile^ and tRNA^ala^ genes respectively. Note that the organization differs somewhat from that described previously by Yu *et al*. (1998) in that Msmeg0011 is clearly part of an operon with Msmeg0012 (*fxuC*), Msmeg0013 (*fxuB*) and Msmeg0014 (*fxuA*). Putative IdeR iron boxes are shown as grey boxes. The lower panel shows the intergenic sequence between Msmeg0010 and Msmeg0011 that contains the putative regulatory features. The iron box consensus recognized by the IdeR regulator is shown above the location of a putative IdeR binding site (bold) upstream of Msmeg0011. The correspondence of this putative site to the consensus sequence (13 of the 19 positions are conserved) is similar to that for the known IdeR binding site upstream of *fxbA* (Msmeg0015) (Dussurget *et al*., 1996), and the position relative to the gene start site and putative −10 and −35 promoter is very similar. Arrows indicate the location of an imperfect inverted repeat that is a potential binding site for a second regulatory protein.

All three operons are iron-responsive, and in planktonic growth, are further repressed (2- to 3-fold) in high-iron conditions (50 μM) relative to either of the low-iron (0 or 2 μM) conditions (Fig. 4A). The overall patterns of response of the three operons during biofilm growth are similar, although the levels of induction are different. Maximal induction is seen at the 4 day point, and when grown in 2 μM iron, Msmeg0012 is induced about 4-fold, Msmeg0015 about 6-fold, and Msmeg4509 about 14-fold. These induction levels are similar in the absence of supplemental iron, although we note that in each case, the induction is somewhat higher in the 3 day samples in the absence of iron than with 2 μM iron (Fig. 4A). Perhaps the most striking observation is that high-iron conditions (50 μM) leads to strong repression of all three operons during biofilm growth (Fig. 4A), consistent with the suppression of the ΔMsmeg0011–0014 and ΔMsmeg0015 mutant defects in high iron (Fig. 3B).

An IdeR binding site (‘iron box’) has previously been identified immediately upstream of the *fxbA* gene (Msmeg0015), and IdeR binding to this site mediates the iron response of this gene (Dussurget *et al*., 1996). Because the *fxu* genes (Msmeg0011–0014) are also iron-responsive, we have examined the region immediately upstream of the first gene of the operon, Msmeg0011 for potential regulatory elements (Fig. 4B). There is only 42 bp in the intergenic region between Msmeg0010 (encoding tRNA-ala) and Msmeg0011, although this contains a putative IdeR binding site and putative promoter sequences. It thus seems likely that IdeR also mediates the iron response of this operon. Because it had been shown previously that *M. smegmatis* siderophore production is also under FurA (Msmeg3466) control (Zahrt *et al*., 2001), we also examined this region for a potential FurA binding site. While we could identify an imperfect inverted repeat (7 of 11 conserved nucleotides) in this region (Fig. 4B), it does not obviously correspond to a FurA binding site (Sala *et al*., 2003).

### Exochelins mediate iron uptake for sliding motility of *M. smegmatis*

**Fig. 5:**
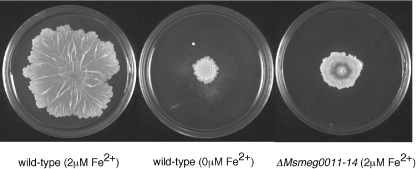
Iron is required for sliding motility of *M. smegmatis*. Colonies of either the parent strain (mc^2^155) or the iron uptake mutant (Δ*Msmeg0011–0014*) were placed at the centre of a sliding agar plate containing either 2 μM iron supplement or no iron (as indicated) and incubated at 37°C. Plates were photographed after 7 days.

It has been shown previously that there is a close correlation between *M. smegmatis* surface motility and biofilm formation. For example, *M. smegmatis* glycopeptidolipid (GPL) mutants are strongly defective in motility and biofilm formation (Martinez *et al*., 1999; Recht *et al*., 2000). We therefore asked whether a 2 μM iron supplement is required for sliding motility, similarly to the requirement for biofilm formation, and observed that motility is strongly reduced when the iron supplement is withdrawn (Fig. 5). Furthermore, we also tested the sliding motility of the mutant defective in exochelin-dependent iron acquisition, ΔMsmeg0011–0014, and found it to be defective even in the presence of 2 μM iron (Fig. 5). These data support the strong linkage between the genetic and physiological requirements for surface motility and biofilm formation.

### Role of iron in biofilm formation and synthesis of C_56_–C_68_ fatty acids

**Fig. 6:**
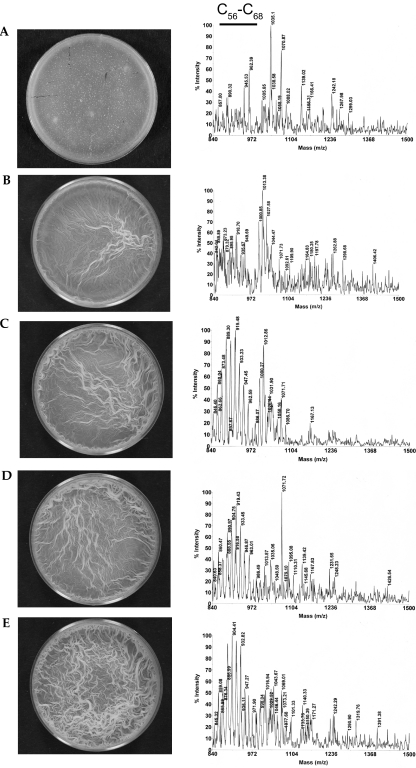
Requirements of supplemental iron for biofilm formation and synthesis of C_56_–C_68_ fatty acids. *M. smegmatis* biofilms were grown for 4 days, with supplemental ferric iron induced in the following concentrations: none (A), 0.5 μM (B), 1 μM (C), 2 μM (D) and 5 μM (E). Samples were harvested, mycolic acids extracted, and analysed by MALDI-TOF as shown in the right. The position of the series of C_56_–C_68_ fatty acids is indicated above.

The microarray and mutant data described above suggest a specific role of iron in biofilm development distinct from that for planktonic growth. To further define this role for iron, we evaluated biofilm formation at intervals of iron concentration between those that do (2 μM) and do not (0 μM) support biofilms (Fig. 6). Because *M. smegmatis* biofilms characteristically contain a novel series of C_56_–C_68_ fatty acids (Ojha *et al*., 2005), we also examined the correlation between iron dependence and the synthesis of these fatty acids (Fig. 6). Visual examination of the dishes shows that addition of supplemental iron at 0.5 μM is sufficient to support only very limited biofilm maturation, and the textured appearance of the biofilms increase as iron concentration increases (Fig. 6); measurements of the accumulated biomass confirm this observation. The synthesis of the C_56_–C_68_ fatty acids closely mirrors this, and they are absent or at low levels in the absence of iron and at 0.5 μM, but are the most prominent fatty acid components at 1 μM and higher iron concentrations (Fig. 6). There is clearly a strong association between supplemental iron concentration and this fatty acid synthesis.

**Fig. 7:**
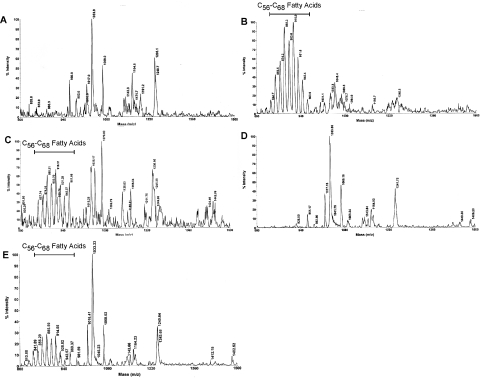
MALDI-TOF analysis of mycolic acid extracts from *M. smegmatis* cells in different growth states. The origin and treatment of cell cultures is as follows. A. Exponential planktonic growth, total-cell extract. B. Four-day biofilm, total-cell extract. C. Stationary-phase culture, total-cell extract. D. Four-day biofilm, cell-associated extract. E. Four-day biofilm, solvent extractable extract.

Because the synthesis of the C_56_–C_68_ fatty acids correlates closely with biofilm maturation, it is likely that these are a central component of the extracellular matrix. To test this, we asked whether these can be extracted from a mature biofilm sample, or whether they remain attached to the cell surface. It is clear that most of these are indeed extractable and thus are presumably matrix components (Fig. 7). We also note that some synthesis of these fatty acids also occurs in stationary-phase cells (Fig. 7).

### Other transcriptomic responses to biofilm formation

The global changes in gene expression during biofilm formation shown in Fig. 2A are likely to include other genes that respond to iron deprivation, as well as those that are either playing a regulatory role in biofilm development, or are perhaps involved in the biosynthesis of the fatty acid matrix material. Altogether, there are more than 100 genes that are significantly induced in biofilms but not in stationary phase (Fig. 2A; Table S2), suggesting a possible biofilm-specific role. These include several genes that are closely associated with *M. tuberculosis* virulence in mice such as an isocitrate lyase, *aceA* (Msmeg0904) (Honer Zu Bentrup *et al*., 1999), transcriptional regulators LexA (Msmeg2743) and Msmeg6566, and many genes involved in transport or uptake of nutrients. Interestingly, there are also indications of at least some cells in the population undergoing an SOS response since not only is LexA induced, but so is UvrD (Msmeg1946) and, to a lesser extent, UvrC (Msmeg3087), RecA (Msmeg2725) and RecX (Msmeg2726) (Table S1). Curiously, while most of the ribosomal subunit genes are expressed at reasonably high levels during exponential planktonic growth (Table S2) and are not induced in biofilm growth, one operon (Msmeg6027–6030) is expressed at only low levels in exponential planktonic growth, but is induced in both biofilm samples and in stationary phase (Tables S1 and S2). This operon encodes the ribosomal proteins S14, S18, L33 and L28 respectively, and is immediately upstream of the iron-acquisition operon Msmeg6024–6026 that is induced similarly (see above). The specific role of these ribosomal proteins is unclear, although we note that there are second copies of each of these genes distributed elsewhere around the *M. smegmatis* genome (Msmeg2399, Msmeg1332, Msmeg1465 and Msmeg6856 respectively), none of which are regulated under any of the tested conditions. It is plausible that these ribosomal proteins are acting as storage units (Panina *et al*., 2003), although we note that other iron-storage proteins, such as bacterioferritins (Msmeg3564, Msmeg6385), are not induced under any of the conditions tested here.

It has been shown previously that GPL synthesis is required for *M. smegmatis* biofilm formation (Recht and Kolter, 2001). The microarray analysis shows that the previously identified gene encoding a peptide synthase, *mps* (Msmeg0390) (Billman-Jacobe *et al*., 1999), is expressed at a moderate level in planktonic growth but is neither induced nor repressed in biofilm formation (Table S1). Interestingly, several of the putative GPL modification enzymes (encoded by genes Msmeg0381–0386) (Patterson *et al*., 2000; Recht *et al*., 2000; Recht and Kolter, 2001; Jeevarajah *et al*., 2002) are substantially downregulated (3- to 8-fold) in biofilm development, including the acetyltransferase, *atf1* (Msmeg0383) that, mutagenesis studies show, is required for normal biofilm formation (Recht and Kolter, 2001). The change in expression of these enzymes suggests the possibility that the GPLs in *M. smegmatis* biofilms may be structurally distinct from those in planktonic growth. Structural variations of GPL in response to limited glucose availability has been previously reported (Ojha *et al*., 2002).

**Fig. 8:**
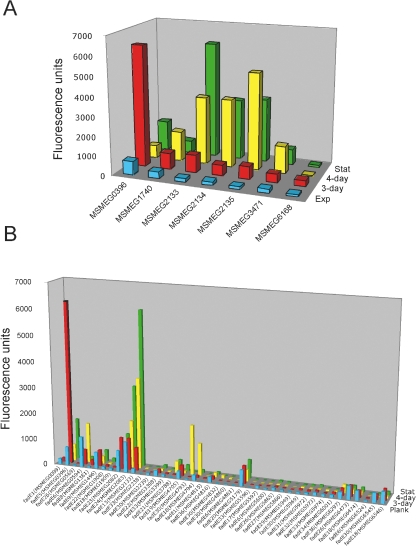
Response of fatty acid metabolism genes during biofilm formation and stationary phase. A. Of the 315 genes putatively involved in fatty acid metabolism, only seven clearly show induced expression (> 4-fold) in either the 3 or 4 day biofilm sample. Average fluorescence values of the seven genes are shown in exponential planktonic growth (blue bars), 3 day biofilms (red bars), 4 day biofilms (yellow bars) and stationary-phase cultures (green bars). Fluorescence values for the exponential planktonic sample are the average of 12 normalized slides, and for the other three experimental conditions, are the average from three independent experiments. B. *M. smegmatis* contains 42 putative *fadE* genes, most of which are not expressed under any of the conditions tested. Average fluorescence values for these 42 genes in exponential planktonic growth (blue bars), 3 day biofilms (red bars), 4 day biofilms (yellow bars) and stationary phase (green bars) are shown. The average values for each condition were calculated as described in panel A.

While the synthesis of the C_56_–C_68_ fatty acids in biofilm development is clear (Fig. 6), the transcriptomic analysis does not provide any clear indications as to which genes are involved. While 315 genes (∼5% of the genome) with putative roles in fatty acid and phospholipid metabolism are identified in the TIGR genome annotation, only a very small proportion undergo substantial changes in expression during biofilm formation (Table S1), and just seven genes in five separate operons are clearly induced more than fourfold in either the 3 or 4 day biofilm sample (Fig. 8). One of these operons (genes Msmeg2133, Msmeg2134 and Msmeg2135; encoding FadE, FadD and an acyl carrier protein respectively) is primarily involved in mycobactin acylation (LaMarca *et al*., 2004) as discussed above, and thus unlikely to play a significant role in matrix synthesis. Msmeg0396 encodes a putative FadE protein that is presumably involved in fatty acid metabolism, and is highly upregulated in the 3 day biofilm sample but not in either the 4 day or stationary-phase samples (Fig. 8A), and thus is also not likely to participate in matrix biosynthesis. Curiously Msmeg0396 is only one of 42 annotated *fadE* genes in the *M. smegmatis* genome, and we have examined the expression patterns of these genes (Fig. 8B), which show that most are either not expressed or expressed at only low levels during exponential growth, and that in the 3 day biofilm sample, Msmeg0396 is expressed at a level higher than any of the other *fadE* genes at any other growth stage. Deletion of Msmeg0396, however, shows no apparent defect in biofilm formation (Table 1). Msmeg 1740 encodes a putative fatty acid desaturase (DesA3), and is upregulated in the 4 day biofilm sample and also to a lesser extent in the 3 day biofilm and stationary-phase samples. Msmeg3471 and Msmeg6168 (encoding an acyl-CoA synthase and a palmitoyl-CoA hydrolase respectively) show modest upregulation in the 4 day biofilm sample, although the overall level of expression is low (Fig. 8A). The role of these in matrix synthesis remains in doubt, and the biosynthesis of the C_56_–C_68_ fatty acids and the correlation to iron availability will be the subject of further study.

## Discussion

While much is known about iron regulation and iron acquisition in mycobacteria, it is clear that many unanswered questions remain. The studies reported here add a critical role of iron in biofilm development to the many different roles that have previously been reported. Just as iron acquisition is necessary for growth of *M. tuberculosis in vitro* and *in vivo* (De Voss *et al*., 2000), it is critical for biofilm formation of *M. smegmatis* in a manner that is distinct from the needs in planktonic growth. Indeed, there appears to be sufficient residual iron in the medium without adding an iron supplement that *M. smegmatis* can readily access enough for planktonic growth with only modest expression of the siderophore biosynthetic genes. In contrast, addition of > 1 μM iron is needed for full biofilm development, and satisfying the iron requirement requires induction of genes involved in exochelin synthesis and uptake of the exochelin–iron complexes. The specific mechanism underlying the biofilm-specific iron demand remains unclear, but we note that iron availability correlates with synthesis of the C_56_–C_68_ fatty acid matrix components, which is likely a major metabolic demand for biofilm maturation, and we speculate that iron plays a role in this process.

Mutational analysis shows clearly that the exochelin system plays a major role in acquiring the levels of iron that are required for biofilm development. Interestingly, the Msmeg0011–0014 operon encoding the exochelin uptake components is induced at the 4 day biofilm stage, although the levels of expression are 2- and 4-fold lower compared with the exochelin and mycobactin biosynthesis genes respectively (Fig. 4A). Presumably, the total amount of iron acquired is regulated by the amount of the uptake proteins available, which are limiting relative to the extracellular siderophores. How the Msmeg0011–0014 operon is regulated in this manner is not clear, although the presence of a putative iron box upstream of Msmeg0011 suggests that IdeR is involved. There may be additional levels of control, and we note that there is at least one additional quasi-symmetric sequence in the small Msmeg0010–Msmeg0011 intergenic region that could represent the binding site for another regulatory protein.

The role of iron in *M. smegmatis* biofilm development shares similarities with the role of iron in biofilm formation by *Pseudomonas aeruginosa* (Banin *et al*., 2005), but also some important distinctions. High iron concentrations promote *P. aeruginosa* biofilm formation (Berlutti *et al*., 2005), while chelation with lactoferrin is strongly inhibitory (Singh *et al*., 2002). However, in contrast to *M. smegmatis*, *P. aeruginosa* biofilms grown in relatively low concentrations of iron shows no substantial difference in expression of the pyoverdine- or pyochelin-dependent iron-acquisition systems as compared with planktonic growth (Whiteley *et al*., 2001). Under low-iron conditions, the pyoverdine system is required for normal biofilm development, and high iron (50 μM) suppresses the mutant defect (Banin *et al*., 2005). A double mutant defective in both the pyoverdine and pyochelin systems is defective in biofilm formation even under high-iron conditions, and the pyochelin system is therefore presumably facilitating iron uptake under these conditions. Presumably the ability of *M. smegmatis* exochelin mutants to grow in high (50 μM) iron is likewise dependent on either the mycobactin or one of the other iron-acquisition transporters. A mutant in the *P. aeruginosa* Fur iron-regulatory protein forms mushroom-like biofilm structures even in the presence of lactoferrin, suggesting that the role of iron is mostly in the regulation of biofilm development (Banin *et al*., 2005). The severe defect in the *M. smegmatis* exochelin uptake mutant suggests that the iron also plays a key role – presumably regulatory in nature – early in biofilm development. However, the correlation of iron availability with C_56_–C_68_ fatty acid synthesis, and requirement for supplemental iron (> 1 μM), suggests that iron may also be needed to satisfy metabolic requirements for the later stages of biofilm maturation.

In other bacteria whose biofilm transcriptome response has been studied, synthesis of the extracellular matrix is accompanied by upregulation of the corresponding biosynthetic genes. *M. smegmatis* appears to be an exception to this, because the fattyome response during biofilm formation is very modest. Moreover, the FAS I and FAS II genes for synthesis of mycolic acids, the most abundant fatty acids in mycobacteria, are constitutively expressed at a high level in both planktonic and biofilm growth. In fact, the greatest responses are observed with genes involved in acylation of the mycobactins (Msmeg2133–2135) and with Msmeg0396, whose induction is largely confined to the 3 day biofilm sample. It therefore seems likely that synthesis of the matrix in this system relies on post-transcriptional modification of the fatty acid synthesis machinery. This is consistent with previous observations showing that the GroEL1 chaperone plays a key role in biofilm maturation and is required for induction of the C_56_–C_68_ series of fatty acids (Ojha *et al*., 2005). This transcriptome analysis shows that neither GroEL1 (Msmeg1581) nor KasA (Msmeg4334) – the protein with which it interacts during biofilm maturation – undergoes any significant change in expression during biofilm formation. In contrast, Msmeg4308, a protein found associated with GroEL1 in mature biofilms (Ojha *et al*., 2005), is induced more than 10-fold in both the 4 day biofilm and stationary-phase samples. Because GroEL1 contains a histidine-rich C-terminus that promotes binding to nickel-affinity columns, an intriguing possibility is that iron plays a direct role in modulating the GroEL1-mediated switch in fatty acid synthesis.

*Mycobacterium smegmatis* induces a number of genes in both biofilms and stationary growth phases that are involved in stress management. For example, the universal stress response proteins Msmeg3816, Msmeg3950 and Msmeg3957 are induced in at least one of the biofilm samples. We also note that *lexA* and *radA* (Msmeg2743 and Msmeg6041 respectively) are induced in biofilm formation, suggesting the possibility that they are responding to oxidative damage to DNA. This could also be associated with the requirement for iron uptake, with O_2_^2–^ and F^2+^ interacting to generate hydroxyl radicals in a Fenton's reaction (Andrews *et al*., 2003). An association between *ideR*-mediated regulation of iron uptake and oxidative stress in *M. smegmatis* is also evident from previous studies (Dussurget *et al*., 1996).

The role of iron in *M. smegmatis* biofilm formation may have implications for the role of iron in the virulence of *M. tuberculosis*, although we note that *M. tuberculosis* does not synthesize exochelin-like siderophores and relies on the mycobactins for iron acquisition. Preliminary observations show that *M. tuberculosis* biofilm formation is also iron-dependent, and we predict that an active mycobactin pathway will be required for this process.

## Experimental procedures

### Bacterial strains, media and growth conditions

*Mycobacterium smegmatis*, *mc*^*2*^*155*, was used as a parent wild-type strain for all the experiments. Bacteria were maintained in 7H10 agar containing appropriate antibiotics −100 ug ml^−1^ carbenicilline, 100 ug ml^−1^ cyclohexamide for the wild type and 150 ug ml^−1^ hygromycine for the mutants. For the microarray experiments, bacteria were grown either as biofilms or as planktonic cultures in the modified M63 media as described earlier (Ojha *et al*., 2005). Planktonic cultures were prepared by inoculating a 25 ml culture of modified M63 medium without tween-80 with 25 μl of a saturated culture grown in 7H9ADCTw broth, and incubated with shaking at 30°C. Biofilm cultures were prepared by inoculation of 10 ml of modified M63 medium with a 10 μl of saturated culture in a 60 × 15 mm polystyrene plastic dish, and incubated at 30°C without shaking for either 3 or 4 days. This modified M63 medium typically contains ferric sulphate at a final concentration of 2 μM. Growth of bacteria on CAS-agar was carried out as described earlier (Fiss *et al*., 1994).

### Microarray analysis

#### 

##### RNA isolation

Bacteria were grown in modified M63 media either as biofilms or planktonically to the desired time or density, harvested and immediately processed for RNA isolation using Ribopure RNA isolation Kit (Ambion). The RNA was analysed for quality by Bioanalyzer (Agilent Technology) and quantified using a NanoDrop Spectrophotmeter before being frozen at −80°C for later use.

##### Labeling and hybridization

A total of 3 μg of total RNA from each culture was reverse transcribed, labelled and hybridized against whole-genome microarrays of *M. smegmatis* according to protocols for two-colour hybridization prepared at TIGR (http://pfgrc.tigr.org/protocols/protocols.shtml). Experiments with each pair of cultures were repeated three times with RNA samples isolated from independent bacterial cultures; the cy3/cy5 dye combination was flipped between the reference and experimental sample in one of the three experiments. Hybridization with cy3- and cy5-labelled pairs of biologically independent reference (exponential planktonic) cultures were used as control for determining the probability score for the genes which showed significant differential expression in experimental (3 day, 4 day and stationary-phase planktonic) samples.

##### Scanning and image processing

Microarray slides upon hybridization were scanned with a dual-channel scanner (Axon) for both cy3 and cy5. The images were then processed by GenePix Pro 4.1. The normalized data were imported into Microsoft Excel for further analysis.

##### Data analysis

Expression data for every gene were screened at several levels before selecting genes for further analysis. First, the genes that had an average of the four median fluorescence values after background subtraction less than 100 in both cy3 and cy5 in all the three replicates of an experiment were removed. Among the genes selected after first screening, only those that had an average log_2_(cy5/cy3) ≥ |±2| in any of the three replicates and *P*-value of ≤ 0.05 when compared with the self-hybridized reference sample were selected for further analysis, as shown in Fig. 2A and Table S2.

## Real-time RT-PCR

Samples containing 800 ng of total RNA from each of the three experimental conditions (3 day biofilm, 4 day biofilm and planktonic stationary phase) and the reference (planktonic exponential phase) were reverse transcribed in a total volume of 100 μl using the cDNA Archive kit (Applied Biosystems) according to the manufacturer's instructions. The 25 μl final reaction mixture of 2.5 μl of reverse transcription reaction, 12.5 μl of SYBR Green Master Mix (Applied Biosystem) and 1.25 nmol each of forward and reverse primers was amplified in Applied Biosystems real-time PCR instrument with the cycle conditions as follows: 50°C, 2 min, 1 cycle; 95°C, 12 min, 1 cycle; 95°C, 15 s; and 60°C, 1 min, 40 cycles. The data were analysed by associated software sds (version 1.1). A gene that showed no obvious regulation (Msmeg2522) in the microarray experiment was used as an endogenous control for normalizing the amount of template in all the experimental conditions. The primer sequences for each of the six ORFs and the endogenous control are provided in Table S3.

## Construction of mutants and biofilm assays

*Mycobacterium smegmatis* mutants were constructed in the wild-type parent strain mc^2^155, by mycobacterial recombineering (van Kessel and Hatfull, 2007). Briefly, the allelic exchange substrate (AES) for every ORF was constructed by PCR amplification of approximately 500 bp corresponding to the upstream and downstream regions and cloning these into the pYUB854 (Bardarov *et al*., 2002) at cloning sites so as to flank the hygromycin-resistance gene. The primers used for amplifying the left arm and the right arm of the AES for every gene are listed in Table S4. The biomass of each of the biofilm cultures was determined by measuring the wet weight of the material scraped out from the air–medium interface.

## Mass spectrometric analysis of mycolic acid extracts

Mycolic acid fractions from respective cultures were extracted and analysed by MALDI-TOF as described previously (Ojha *et al*., 2005). Mycolates from total cellular, extractable and cell-bound lipids were extracted by treating the samples overnight with 2 ml tetrabutylammonium hydroxide (20%) at 100°C, addition of 2 ml of dichloromethane and 200 ml of methyl iodide, and mixing at room temperature for 1 h. After centrifugation at 2000 r.p.m. for 10 min, the organic phase was washed once with 0.25 N HCl and once with water before drying under vacuum. The extractable lipids were separated from the cell-bound lipid by treating the cells with 4 ml of chloroform/methanol/water (10:10:3) at 37°C overnight, washing the organic layer first with 0.75 ml chloroform/1.75 ml water and then twice with 2 ml of chloroform/methanol/water (3:47:48). The lower organic layer yielded the extractable lipid.

## Supplementary material

This material is available as part of the online article from: http://www.blackwell-synergy.com/doi/abs/10.1111/j.1365-2958.2007.05935.x (This link will take you to the article abstract).

## References

[b1] Andrews SC, Robinson AK, Rodriguez-Quinones F (2003). Bacterial iron homeostasis. FEMS Microbiol Rev.

[b2] Banin E, Vasil ML, Greenberg EP (2005). Iron and *Pseudomonas aeruginosa* biofilm formation. Proc Natl Acad Sci USA.

[b3] Bardarov S, Bardarov S, Pavelka MS, Sambandamurthy V, Larsen M, Tufariello J (2002). Specialized transduction: an efficient method for generating marked and unmarked targeted gene disruptions in *Mycobacterium tuberculosis*, *M. bovis* BCG and *M. smegmatis*. Microbiology.

[b4] Beenken KE, Dunman PM, McAleese F, Macapagal D, Murphy E, Projan SJ (2004). Global gene expression in *Staphylococcus aureus* biofilms. J Bacteriol.

[b5] Beloin C, Ghigo JM (2005). Finding gene-expression patterns in bacterial biofilms. Trends Microbiol.

[b6] Beloin C, Valle J, Latour-Lambert P, Faure P, Kzreminski M, Balestrino D (2004). Global impact of mature biofilm lifestyle on *Escherichia coli* K-12 gene expression. Mol Microbiol.

[b7] Berlutti F, Morea C, Battistoni A, Sarli S, Cipriani P, Superti F (2005). Iron availability influences aggregation, biofilm, adhesion and invasion of *Pseudomonas aeruginosa* and *Burkholderia cenocepacia*. Int J Immunopathol Pharmacol.

[b8] Billman-Jacobe H, McConville MJ, Haites RE, Kovacevic S, Coppel RL (1999). Identification of a peptide synthetase involved in the biosynthesis of glycopeptidolipids of *Mycobacterium smegmatis*. Mol Microbiol.

[b9] Carter G, Wu M, Drummond DC, Bermudez LE (2003). Characterization of biofilm formation by clinical isolates of *Mycobacterium avium*. J Med Microbiol.

[b10] Cho KH, Caparon MG (2005). Patterns of virulence gene expression differ between biofilm and tissue communities of *Streptococcus pyogenes*. Mol Microbiol.

[b11] Costerton JW, Stewart PS, Greenberg EP (1999). Bacterial biofilms: a common cause of persistent infections. Science.

[b12] Davey ME, O'Toole GA (2000). Microbial biofilms: from ecology to molecular genetics. Microbiol Mol Biol Rev.

[b13] De Voss JJ, Rutter K, Schroeder BG, Barry CE (1999). Iron acquisition and metabolism by mycobacteria. J Bacteriol.

[b14] De Voss JJ, Rutter K, Schroeder BG, Su H, Zhu Y, Barry CE (2000). The salicylate-derived mycobactin siderophores of *Mycobacterium tuberculosis* are essential for growth in macrophages. Proc Natl Acad Sci USA.

[b15] Domka J, Lee J, Bansal T, Wood TK (2007). Temporal gene-expression in *Escherichia coli* K-12 biofilms. Environ Microbiol.

[b16] Dussurget O, Rodriguez M, Smith I (1996). An ideR mutant of *Mycobacterium smegmatis* has derepressed siderophore production and an altered oxidative-stress response. Mol Microbiol.

[b17] Fiss EH, Yu S, Jacobs WR (1994). Identification of genes involved in the sequestration of iron in mycobacteria: the ferric exochelin biosynthetic and uptake pathways. Mol Microbiol.

[b18] Frieden TR, Driver CR (2003). Tuberculosis control: past 10 years and future progress. Tuberculosis (Edinb).

[b19] Gomez JE, McKinney JD (2004). *M. tuberculosis* persistence, latency, and drug tolerance. Tuberculosis (Edinb).

[b20] Hall-Stoodley L, Lappin-Scott H (1998). Biofilm formation by the rapidly growing mycobacterial species *Mycobacterium fortuitum*. FEMS Microbiol Lett.

[b21] Hall-Stoodley L, Costerton JW, Stoodley P (2004). Bacterial biofilms: from the natural environment to infectious diseases. Nat Rev Microbiol.

[b22] Hall-Stoodley L, Brun OS, Polshyna G, Barker LP (2006). *Mycobacterium marinum* biofilm formation reveals cording morphology. FEMS Microbiol Lett.

[b23] Honer Zu Bentrup K, Miczak A, Swenson DL, Russell DG (1999). Characterization of activity and expression of isocitrate lyase in *Mycobacterium avium* and *Mycobacterium tuberculosis*. J Bacteriol.

[b24] Jeevarajah D, Patterson JH, McConville MJ, Billman-Jacobe H (2002). Modification of glycopeptidolipids by an O-methyltransferase of *Mycobacterium smegmatis*. Microbiology.

[b25] van Kessel JC, Hatfull GF (2007). Recombineering in *Mycobacterium tuberculosis*. Nat Methods.

[b26] Krithika R, Marathe U, Saxena P, Ansari MZ, Mohanty D, Gokhale RS (2006). A genetic locus required for iron acquisition in *Mycobacterium tuberculosis*. Proc Natl Acad Sci USA.

[b27] LaMarca BB, Zhu W, Arceneaux JE, Byers BR, Lundrigan MD (2004). Participation of fad and mbt genes in synthesis of mycobactin in *Mycobacterium smegmatis*. J Bacteriol.

[b28] Lazazzera BA (2005). Lessons from DNA microarray analysis: the gene expression profile of biofilms. Curr Opin Microbiol.

[b29] McCune RM, Feldmann FM, Lambert HP, McDermott W (1966). Microbial persistence. I. The capacity of tubercle bacilli to survive sterilization in mouse tissues. J Exp Med.

[b30] Marsollier L, Aubry J, Coutanceau E, Andre JP, Small PL, Milon G (2005). Colonization of the salivary glands of *Naucoris cimicoides* by Mycobacterium ulcerans requires host plasmatocytes and a macrolide toxin, mycolactone. Cell Microbiol.

[b31] Martinez A, Torello S, Kolter R (1999). Sliding motility in mycobacteria. J Bacteriol.

[b32] Milano A, Forti F, Sala C, Riccardi G, Ghisotti D (2001). Transcriptional regulation of furA and katG upon oxidative stress in *Mycobacterium smegmatis*. J Bacteriol.

[b33] Moorthy S, Watnick PI (2005). Identification of novel stage-specific genetic requirements through whole genome transcription profiling of *Vibrio cholerae* biofilm development. Mol Microbiol.

[b34] O'Toole G, Kaplan HB, Kolter R (2000). Biofilm formation as microbial development. Annu Rev Microbiol.

[b35] Ojha A, Anand M, Bhatt A, Kremer L, Jacobs WR, Hatfull GF (2005). GroEL1: a dedicated chaperone involved in mycolic acid biosynthesis during biofilm formation in mycobacteria. Cell.

[b36] Ojha AK, Varma S, Chatterji D (2002). Synthesis of an unusual polar glycopeptidolipid in glucose-limited culture of *Mycobacterium smegmatis*. Microbiology.

[b37] Panina EM, Mironov AA, Gelfand MS (2003). Comparative genomics of bacterial zinc regulons: enhanced ion transport, pathogenesis, and rearrangement of ribosomal proteins. Proc Natl Acad Sci USA.

[b38] Patterson JH, McConville MJ, Haites RE, Coppel RL, Billman-Jacobe H (2000). Identification of a methyltransferase from *Mycobacterium smegmatis* involved in glycopeptidolipid synthesis. J Biol Chem.

[b39] Pruss BM, Besemann C, Denton A, Wolfe AJ (2006). A complex transcription network controls the early stages of biofilm development by *Escherichia coli*. J Bacteriol.

[b40] Pysz MA, Conners SB, Montero CI, Shockley KR, Johnson MR, Ward DE, Kelly RM (2004). Transcriptional analysis of biofilm formation processes in the anaerobic, hyperthermophilic bacterium *Thermotoga maritima*. Appl Environ Microbiol.

[b41] Recht J, Kolter R (2001). Glycopeptidolipid acetylation affects sliding motility and biofilm formation in *Mycobacterium smegmatis*. J Bacteriol.

[b42] Recht J, Martinez A, Torello S, Kolter R (2000). Genetic analysis of sliding motility in *Mycobacterium smegmatis*. J Bacteriol.

[b43] Ren D, Bedzyk LA, Thomas SM, Ye RW, Wood TK (2004a). Gene expression in *Escherichia coli* biofilms. Appl Microbiol Biotechnol.

[b44] Ren D, Bedzyk LA, Setlow P, Thomas SM, Ye RW, Wood TK (2004b). Gene expression in *Bacillus subtilis* surface biofilms with and without sporulation and the importance of yveR for biofilm maintenance. Biotechnol Bioeng.

[b45] Rodriguez GM, Smith I (2003). Mechanisms of iron regulation in mycobacteria: role in physiology and virulence. Mol Microbiol.

[b46] Rodriguez GM, Voskuil MI, Gold B, Schoolnik GK, Smith I (2002). ideR, an essential gene in mycobacterium tuberculosis: role of IdeR in iron-dependent gene expression, iron metabolism, and oxidative stress response. Infect Immun.

[b47] Rose L, Kaufmann SH, Daugelat S (2004). Involvement of *Mycobacterium smegmatis* undecaprenyl phosphokinase in biofilm and smegma formation. Microbes Infect.

[b48] Sala C, Forti F, Di Florio E, Canneva F, Milano A, Riccardi G, Ghisotti D (2003). *Mycobacterium tuberculosis* FurA autoregulates its own expression. J Bacteriol.

[b49] Sampathkumar B, Napper S, Carrillo CD, Willson P, Taboada E, Nash JH (2006). Transcriptional and translational expression patterns associated with immobilized growth of *Campylobacter jejuni*. Microbiology.

[b50] Schembri MA, Kjaergaard K, Klemm P (2003). Global gene expression in *Escherichia coli* biofilms. Mol Microbiol.

[b51] Singh PK, Parsek MR, Greenberg EP, Welsh MJ (2002). A component of innate immunity prevents bacterial biofilm development. Nature.

[b52] de Souza AA, Takita MA, Coletta-Filho HD, Caldana C, Yanai GM, Muto NH (2004). Gene expression profile of the plant pathogen *Xylella fastidiosa* during biofilm formation *in vitro*. FEMS Microbiol Lett.

[b53] de Souza AA, Takita MA, Pereira EO, Coletta-Filho HD, Machado MA (2005). Expression of pathogenicity-related genes of *Xylella fastidiosa in vitro* and in planta. Curr Microbiol.

[b54] Stanley NR, Britton RA, Grossman AD, Lazazzera BA (2003). Identification of catabolite repression as a physiological regulator of biofilm formation by *Bacillus subtilis* by use of DNA microarrays. J Bacteriol.

[b55] Stewart GR, Robertson BD, Young DB (2003). Tuberculosis: a problem with persistence. Nat Rev Microbiol.

[b56] Teng R, Dick T (2003). Isoniazid resistance of exponentially growing *Mycobacterium smegmatis* biofilm culture. FEMS Microbiol Lett.

[b57] Timm J, Post FA, Bekker LG, Walther GB, Wainwright HC, Manganelli R (2003). Differential expression of iron-, carbon-, and oxygen-responsive mycobacterial genes in the lungs of chronically infected mice and tuberculosis patients. Proc Natl Acad Sci USA.

[b58] Waite RD, Papakonstantinopoulou A, Littler E, Curtis MA (2005). Transcriptome analysis of *Pseudomonas aeruginosa* growth: comparison of gene expression in planktonic cultures and developing and mature biofilms. J Bacteriol.

[b59] Waite RD, Paccanaro A, Papakonstantinopoulou A, Hurst JM, Saqi M, Littler E, Curtis MA (2006). Clustering of *Pseudomonas aeruginosa* transcriptomes from planktonic cultures, developing and mature biofilms reveals distinct expression profiles. BMC Genomics.

[b60] Whiteley M, Bangera MG, Bumgarner RE, Parsek MR, Teitzel GM, Lory S, Greenberg EP (2001). Gene expression in *Pseudomonas aeruginosa* biofilms. Nature.

[b61] Wisedchaisri G, Chou CJ, Wu M, Roach C, Rice AE, Holmes RK (2007). Crystal structures, metal activation, and DNA-binding properties of two-domain IdeR from *Mycobacterium tuberculosis*. Biochemistry.

[b62] Yang YH, Dudoit S, Luu P, Lin DM, Peng V, Ngai J, Speed TP (2002). Normalization for cDNA microarray data: a robust composite method addressing single and multiple slide systematic variation. Nucleic Acids Res.

[b63] Yu S, Fiss E, Jacobs WR (1998). Analysis of the exochelin locus in *Mycobacterium smegmatis*: biosynthesis genes have homology with genes of the peptide synthetase family. J Bacteriol.

[b64] Zahrt TC, Song J, Siple J, Deretic V (2001). Mycobacterial FurA is a negative regulator of catalase-peroxidase gene katG. Mol Microbiol.

[b65] Zambrano MM, Kolter R (2005). Mycobacterial biofilms: a greasy way to hold it together. Cell.

