# Supplementary material for: The role of iron in Mycobacterium smegmatis biofilm formation: the exochelin siderophore is essential in limiting iron conditions for biofilm formation but not for planktonic growth
Source: Mol Microbiol. 2007 Oct;66(2):468–83. doi: 10.1111/j.1365-2958.2007.05935.x (PMC2170428; doi:10.1111/j.1365-2958.2007.05935.x)
Supplement: Supplementary file 1 [file mmi0066-0468-SD1.pdf]

Supplementary Data

Figure S1. Comparison of microarray-based transcriptome responses with Real-Time PCR (RT-PCR).

A. RNA levels of 16 genes (Msmeg3903, Msmeg 3904, Msmeg 4307, Msmeg 4308, Msmeg 4507, Msmeg 4509, Msmeg 5992, Msmeg 6008, Msmeg 6012, Msmeg 6019, Msmeg 6020, Msmeg 6026, Msmeg 6030, Msmeg 6031, Msmeg 6721, Msmeg 6858) were determined by RT-PCR and compared with the microarray data, using 3-day biofilm and exponential phase planktonic cultures. The x-axis represents $\log_2$ ratios of the sixteen genes from microarray experiments whereas y-axis represents the corresponding ΔC_t values from the RT-PCR experiments. Average values from three separate microarray hybridization and three separate RT-PCR experiments are plotted. While the RT-PCR and microarray data are closely correlated (R^2=0.88), the broader linearity of the RT-PCR analysis relative to microarray hybridization results in unequal magnitudes of RNA differences.

B. RNA levels of 4 genes (Smeg0396, Smeg0576, Msmeg0923, and Msmeg1172) were compared by RT-PCR (blue bars) and microarray experiments (maroon bars), using pairs of RNA samples corresponding to exponential planktonic/exponential planktonic (Exp), exponential planktonic/3-day biofilm (3dbf), exponential planktonic/4-day biofilm (4dbf) and exponential planktonic/stationary phase (stphase) comparisons. The $\log_2$ differences for each sample are shown.

Figure S2. Scatter plots of average fluorescence intensities of each of the 6530 spots from three independent hybridizations of reference and experimental samples. Parts A-D correspond to a planktonic versus planktonic control (A), 3-day biofilm versus planktonic reference (B), 4-day biofilm versus planktonic reference (C), and stationary phase versus planktonic reference (D). The regions outside of the red lines correspond to four-fold variance for any given gene. In the control hybridization (A), only 7 genes (<0.1%) have fluorescence values above 100 units in both

samples and vary by more than four-fold. The correlation value $R^2 = 0.88$, and most of the variance likely derives from the inclusion of three separate biological samples that were grown and prepared independently. There is an increased variance in all three of the experimental cases (B-D) which is indicated by an increase in the number of sample points outside of the red line and an decrease in the correlation values (R^2=0.62, 0.37 and 0.44 for panels B-D respectively) reflecting the transcriptomic changes.

Table S1. Table shows the average $\log_2$ ratios of expression levels between experimental samples (3-day, 4-day or stationary phase) and a reference sample (exponential planktonic culture) for all 6530 genes printed on the microarray slides. Each value represents the average of four duplicated spots of a gene in a slide. A self-hybridized reference sample is used as a control for calculating the probability score. The last three columns are the average fluorescence levels for each gene calculated from all three slides representing that particular growth condition. ND denotes values not determined.

Table S2. List of most highly expressed genes during *M. smegmatis* exponential planktonic growth

Table S3. The table includes several lists of genes that are differentially expressed by 4-fold or more in at least one of the three replicate samples with an overall P-score <0.05 in either 3-day biofilm, 4-day biofilm or stationary phase experimental samples, relative to the exponential planktonic sample. Each list corresponds to the numbers of genes shown in Fig. 2A. ND denotes values not determined.

Table S4. List of oligonucleotides used for RT-PCR.

Table S5. List of oligonucleotides used for constructing gene knockout mutants

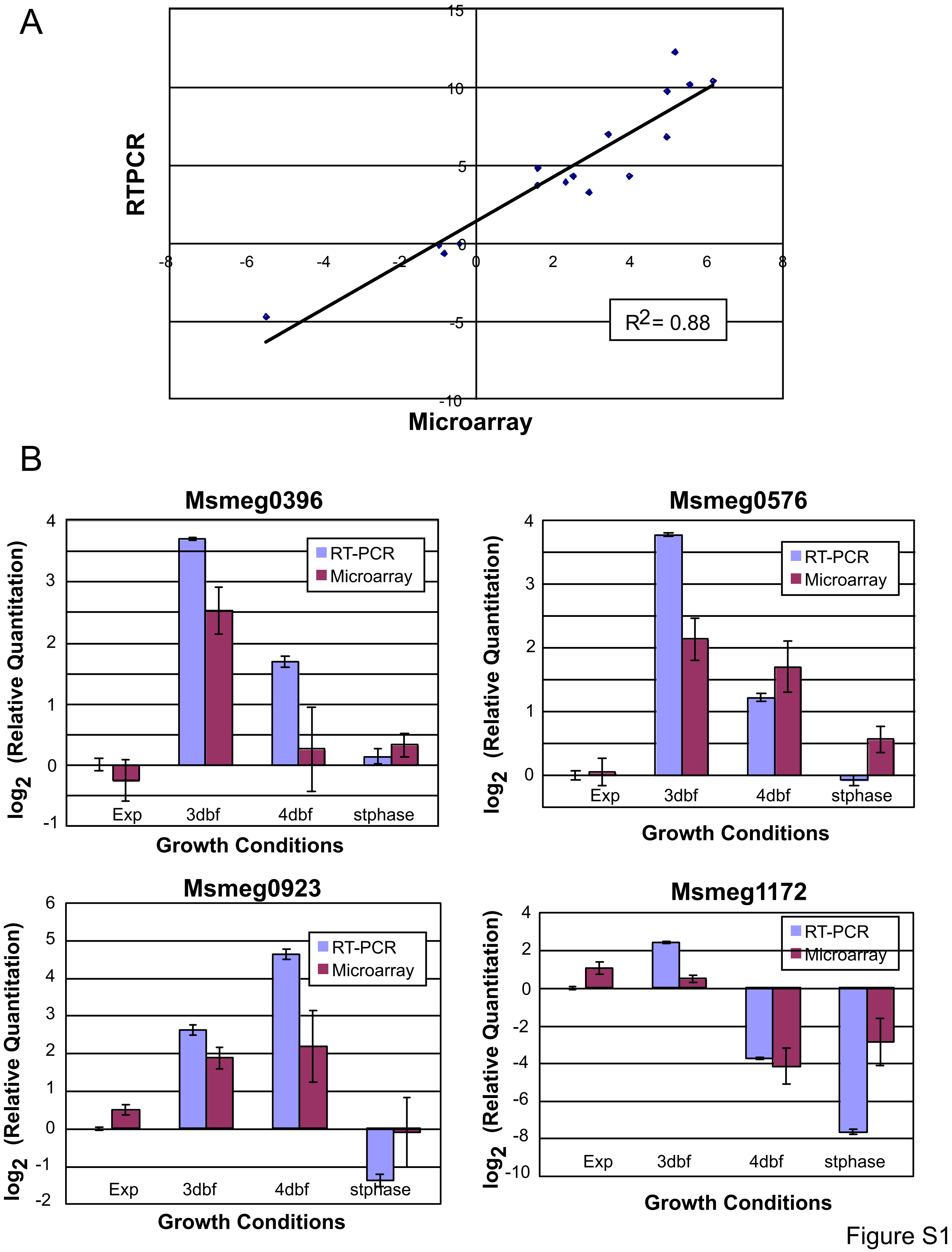
A
RTPCR
Microarray
R^2= 0.88
B
Msmeg0396
Msmeg0576
Msmeg0923
Msmeg1172
RT-PCR
Microarray
log2 (Relative Quantitation)
Exp
3dbf
4dbf
stphase
Growth Conditions

Figure S1

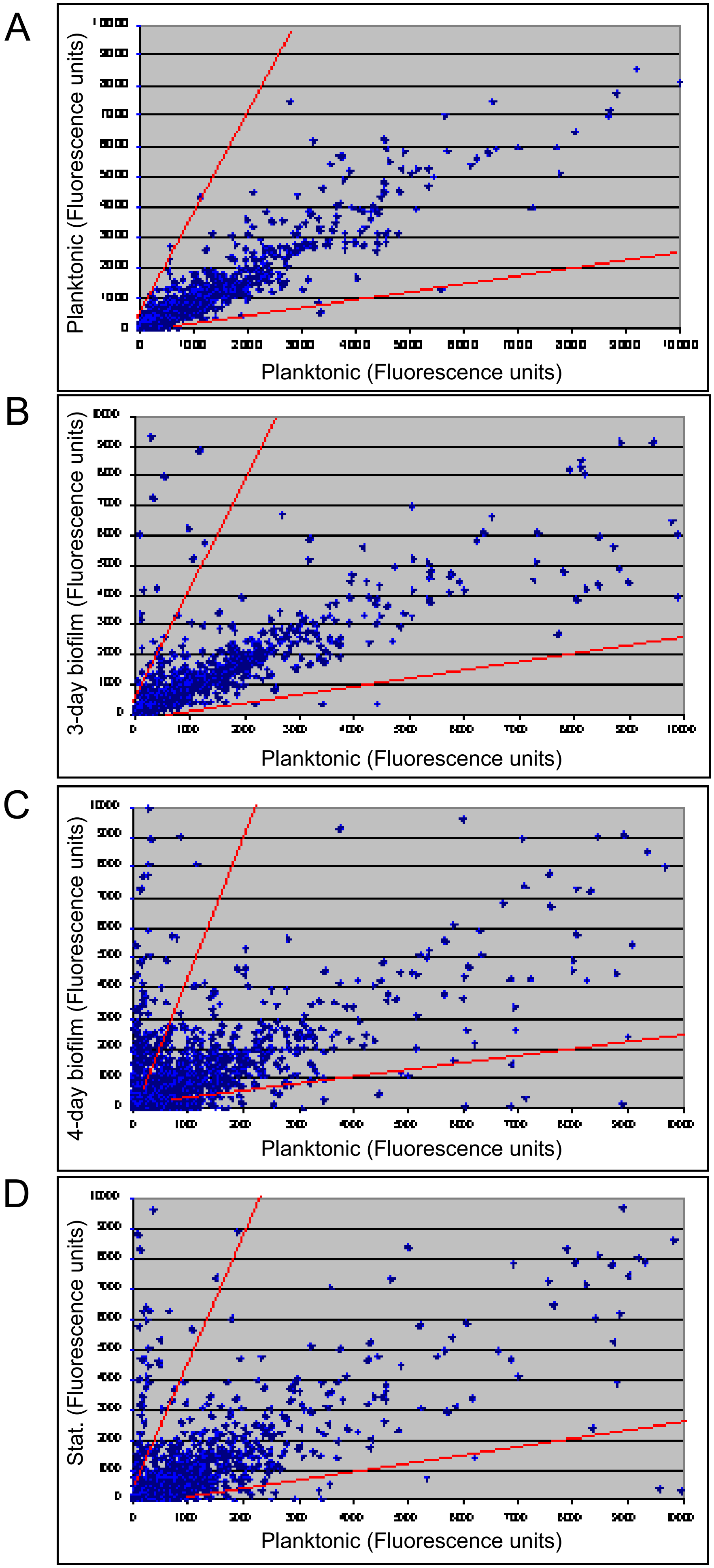
A
Planktonic (Fluorescence units)
Planktonic (Fluorescence units)
B
3-day biofilm (Fluorescence units)
Planktonic (Fluorescence units)
C
4-day biofilm (Fluorescence units)
Planktonic (Fluorescence units)
D
Stat. (Fluorescence units)
Planktonic (Fluorescence units)

Figure S2

Table S2. Most highly expressed *M. smegmatis* genes and operons in exponential planktonic growth

**Genes/Operon**[a]	**# genes in operon**	**Gene Name/Function**	**Expression^**
MSMEG0955	1	mspA-Gen	29081
MSMEG6360	1	esterase, putative, antigen 85-A	19652
MSMEG6719-21	3	glpF, glpK, glpD2	17733
MSMEG6038-9	2	ispD; regulator	15463
MSMEG0873	1	60 kd chaperonin , fragment	15093
MSMEG6390	1	superoxide dismutase	10802
MSMEG1553-4	2	ribosomal proteins	8682
MSMEG5103-4	2	pruA	8084
MSMEG1393-7	5	ribosomal proteins; translation factors	7926
MSMEG1580	1	groES	7427
MSMEG1357-8	2	ribosomal proteins	7250
MSMEG1339-40	2	ribosomal proteins	6718
MSMEG3093-4	2	gap; pgk	6581
MSMEG1434-43	10	ribosomal proteins	6542
MSMEG3624	1	hypothetical protein	6500
MSMEG5663	1	conserved hypothetical protein	6380
MSMEG1675-80	6	aspA; amiB	6350
MSMEG0550[#]	1	cold shock protein	6345
MSMEG0064-5	2	conserved hypothetical proteins	6323
MSMEG1832	1	conserved hypothetical protein	6117
MSMEG6622	1	Oxiforeductase	5825
MSMEG0234[#]	1	conserved hypothetical protein	5560
MSMEG3797-8	2	ribosomal proteins	5515
MSMEG2522	1	ribosomal protein	5458
MSMEG4328	1	aceE	5262
MSMEG3066	1	S-adenosylmethionine synthetase	5059
MSMEG1045	1	lysyl tRNA synthetase-like protein	5046
MSMEG6046	1	transcription regulator	4963
MSMEG1462-71	10	ribosomal proteins	4960
MSMEG2350-1	2	etfA,B	4956
MSMEG2388	1	histone-like protein	4731
MSMEG2298-99	2	glutaredoxin, nrdI	4620
MSMEG0631-4	4	ABC transporters	4508
MSMEG2433	1	ribosomal protein S16	4502

[a]Genes and operons shown represent ~1% of the most highly expressed of genes in planktonic exponential growth
^Expression levels are rank-ordered by fluorescence levels. Average values are shown for multi-gene operons
[#]Operons may contain additional genes not printed on microarray slides

Table S4. List of oligonucleotides used for RT-PCR

Gene name	Forward Primer	Reverse Primer
Msmeg0012	gcttttctcggagcgcttt	cggtcatgccgatcaggta
Msmeg0015	gcaggcgttgttgaaatcg	cgattcgtacgcatggtcact
Msmeg0396	cgcaagatcatccgtgacaa	gccgttctcgttcttgatgaac
Msmeg0576	cgcaagatcatccgtgacaa	gccgttctcgttcttgatgaa
Msmeg0923	gatcgcccgtgatgtcatc	cgtgacctggcggttca
Msmeg1172	aggtgttcgactacggcaactc	gaattgcgggcggatgta
Msmeg1174	gcgtcggcgagttcca	gagcagcgtgatccggatac
Msmeg1739	cgtatacgtctgcggtccaa	actcggatcgcacgttgtc
Msmeg2522	ctgcagcagacgctgaccta	tgggcgacggtttcctt
Msmeg3903	ggtccaagcgcatcatcct	gtgctcgtagcggtactcgtt
Msmeg3904	ccaacgcggtggaatcc	cgtcatgccgtgaaatgc
Msmeg4301	accaatttatggtgctgcaaacac	cggccttccacttctgcaa
Msmeg4307	tccgccctgagcgaatatc	aactcgttcgcgcagatcac
Msmeg4308	cgaccgcatcacattcga	tgcaggaattgcgctgaca
Msmeg4507	gcttcaagcccatcaacga	acgccggatcgaattcac
Msmeg4509	ccggcgaacgaatttcc	cgaagacctcaccctgcaa
Msmeg5285	tgttcgtcaacctgatcaagatg	gaccgtggcggctttg
Msmeg5992	attccgacgtcccttcagaa	gagtggttgaaccaccgatagg
Msmeg6008	caccgttctgtccggcttt	gttggcgaggatgtgattga
Msmeg6012	cggtgagcacagcgatgtc	cggtgagcacagcgatgtc
Msmeg6019	gtcgcctccgtcgatgag	gtcgcctccgtcgatgag
Msmeg6020	ctggtgctgcgcgaaaac	acgtcaggtcctggaggtactc
Msmeg6026	aaaggcatcggcaatcagat	cgtcgccgtagtcgtagatca
Msmeg6030	ccccaatatccagaacaagaccta	cgatcaccttgatgccttttg
Msmeg6031	gacagctctcagtgggtacttgag	cagacggcgaagcaggat
Msmeg6721	accgacgagttgctcgaaa	gtagtacgcctccaccttgagatac
Msmeg6858	cattctcgaccccacacttga	ccttgcggatgacgttcag

Table S5. List of oligonucleotides used for constructing gene knockout mutants

smeg5028kontf	gcggtgctgggccagcttaaggtgcgcggctacgcg
smeg5028kontr	ccggggccgagaagtctagacgcaggtagtggcgtg
smeg5028koctf	ggcatcccgcgccgctagctaaaggcgcaggcctac
smeg5028koctr	cgctttcggtgccgtctcgagccggtgcgtgatggcg
smeg0011kontf	tcatctacaacctcacgacagatctcgt
smeg0011kontr	gccgagtcggcggcctgctagcacgtcgagggaagcgg
smeg0014koctf	atctggtgcgcgacgtctagaggctggaatgccagat
smeg0014koctr	gcatgattcgctgctgcttaagttcaccggtttctcg
smeg6007kontf	cgcgtgccagttcgcttaagaacgtcgagacatcga
smeg6007kontr	gccggctcggccgtctagaattcgtcggagccgcatg
smeg6006koctr	tgtgatcgccagcagatctccgaagagcacgttggt
smeg6006koctf	gcgtgctcgacagcgctagctacctgctcgacgggca
smeg6025kontf	accatcggagagatcttaagggtcgaggagcgcgcg
smeg6025kontr	cacacccgcggtcatctagagcagcaggagcaccacc
smeg6024koctf	ctgaccgtcgagctgctagcggaggtctacggggtc
smeg6024koctr	cgccgcgtcggtgctcgaggtcgccaacgacagcg
smeg3411kontf	gtcggttgccttaagaggtcccgacca
smeg3411kontr	cacgatctctagaatcttcgcgtcgat
smeg3411koctf	cgacagaggaagcttgttgaccaacca
smeg3411koctr	gggctcatcctcgagtacaacgct
smeg5088kontf	catcggtcaccgtcaacttaaggttcgc
smeg5088kontr	cgccgttgggtgcgtctagacccgt
smeg5088koctf	gccatgacgaagctttcctcgacacca
smeg5088koctr	gatgccgaggagatctgggaaccct
smeg0015kontf	taccggacgtgagccacttaagatttcggctcacgg
smeg0015kontr	gggtacgccaattgttctagacgccgacatccggtgc
smeg0015koctf	atgcacctacggtggctagccaggacgtgtgttcatc
smeg0015koctr	ggtatcacggtggcagatctggtgtcgcgtggc
smeg4509kontf	tcgtgttcgggaggccttaagggcgacgtcgctggtgc
smeg4509kontr	agcaaagacagtgtcatctagaagacctcaccctgcaa
smeg4509koctf	cagggaggccgacgctagccgcctcgaacaagtc
smeg4509koctr	ccccttcggtgtagatctgccagccgcgcagcat
smeg0396kontf	cgacatgtttgccagatctcagacagcgctcgatgtca
smeg0396kontr	ggtcgctgaacttgctagcgccgagcaccttgtccac
smeg0396koctf	ggacgaggcggcgttctagaggcgcgttctgag
smeg0396koctr	tcagcgaggtccggcttaagcggctgggcaacctcggc
